# Skeletal carbonate mineralogy of Scottish bryozoans

**DOI:** 10.1371/journal.pone.0197533

**Published:** 2018-06-13

**Authors:** Jennifer Loxton, Mary Spencer Jones, Jens Najorka, Abigail M. Smith, Joanne S. Porter

**Affiliations:** 1 Centre for Marine Biodiversity and Biotechnology, School of Life Sciences, Heriot-Watt University, Riccarton, Edinburgh, Uinted Kingdom; 2 Department of Life Sciences, Natural History Museum, London, Uinted Kingdom; 3 University Marine Biological Station, Millport, Isle of Cumbrae, Uinted Kingdom; 4 Core Research Laboratories, Natural History Museum, London, Uinted Kingdom; 5 Department of Marine Science, University of Otago, Dunedin, New Zealand; University of Minnesota, UNITED STATES

## Abstract

This paper describes the skeletal carbonate mineralogy of 156 bryozoan species collected from Scotland (sourced both from museum collections and from waters around Scotland) and collated from literature. This collection represents 79% of the species which inhabit Scottish waters and is a greater number and proportion of extant species than any previous regional study. The study is also of significance globally where the data augment the growing database of mineralogical analyses and offers first analyses for 26 genera and four families. Specimens were collated through a combination of field sampling and existing collections and were analysed by X-ray diffraction (XRD) and micro-XRD to determine wt% MgCO_3_ in calcite and wt% aragonite. Species distribution data and phylogenetic organisation were applied to understand distributional, taxonomic and phylo-mineralogical patterns. Analysis of the skeletal composition of Scottish bryozoans shows that the group is statistically different from neighbouring Arctic fauna but features a range of mineralogy comparable to other temperate regions. As has been previously reported, cyclostomes feature low Mg in calcite and very little aragonite, whereas cheilostomes show much more variability, including bimineralic species. Scotland is a highly variable region, open to biological and environmental influx from all directions, and bryozoans exhibit this in the wide range of within-species mineralogical variability they present. This plasticity in skeletal composition may be driven by a combination of environmentally-induced phenotypic variation, or physiological factors. A flexible response to environment, as manifested in a wide range of skeletal mineralogy within a species, may be one characteristic of successful invasive bryozoans.

## Introduction

Many marine organisms make carbonate shells and skeletons, which can act as repositories for information about seawater conditions. Biomineralisation can be controlled by the environment (e.g. algae [1] and corals [2,3] but many invertebrates, such as Foraminifera [4] and Mollusca [5], also exert biological control over the calcification process, managing nucleation [6–8], ultrastructure and crystal fabric [9–11], and carbonate composition [12–15]. Calcification can develop along with ontogeny/astogeny (e.g., growth rate [16–18], age [19–21]) or be determined by genetic factors and phylogenetic position [1,22–24]. Even highly controlling organisms, however, may be influenced by environmental parameters such as temperature [16–21], salinity [22–24], and pH [25,26].

Marine bryozoans are colonial filter-feeding invertebrates and are well characterised globally in terms of mineralogy [27]. Unlike some groups, where perhaps 2–3% of species have actually been measured (e.g., 29 chiton species = 3% of extant species [22]), marine bryozoans have been extensively studied at both ends of the Earth [14,15,17–24], such that at least a quarter of extant species have been characterised at least once (~1500 species [28]).

Bryozoan skeletons are mineralogically variable, and can be entirely calcitic, entirely aragonitic or bimineralic, featuring more than one CaCO_3_ polymorph [27,29]. There is also a wide range of Magnesium (Mg) content in bryozoan calcite, ranging from low magnesium carbonate (LMC, < 4 wt% MgCO_3_), through intermediate magnesium carbonate (IMC, >4 wt%MgCO_3_ to < 8 wt%MgCO_3)_ to high magnesium carbonate (HMC, >8 wt%MgCO_3_) [20,27] with most bryozoans featuring low to intermediate Mg-calcite [27,20,29,30]. This variation can be ascribed to environmental, physiological or phylogenetic influences [31].

Despite the strong phylogenetic signal in bryozoan mineralogy [24], it is links between mineralogy and environmental conditions that are the main driving force behind many mineralogical studies. Since the mid twentieth century [32] the skeleton composition of biological groups, such as the Foraminifera [33] and Mollusca [34], have been used in palaeoclimatology, where historic and fossil specimens have been assumed to hold a record of the seawater chemistry and temperature from the time in which it was deposited. Additionally, in recent years, it has been hoped that animals with variable calcium carbonate chemistry, such as the Bryozoa, might be able to act as a “bellweather” for climate change and ocean acidification [35]–with their skeletal chemistry proving that changes in our oceans are having a measureable effect on the biota which inhabit them. It is this spur of climate change and ocean acidification that has fuelled some of the increase in bryozoan mineralogy studies over the past decade.

Since the beginning of mineralogy studies on the Bryozoa there have been region-specific publications. The majority are of limited use due to the very low specimen numbers analysed (e.g. South Africa [36], Hawaii [37], Talbot Shoal [38], Naples [39], Malaysia [40]). There have, however, also been a handful of studies where the coverage of regional bryozoans has been great enough to draw meaningful conclusions. These studies include: the Mediterranean with 94/300 species analysed [41]; Antarctica with 21/300 species analysed [42]; New Zealand with 49/953 species analysed [43]; Chile with 23/267 species analysed [24] and the Arctic with 76/300 species analysed [20].

Scotland was chosen for this regional study for its ecological and biological diversity. Scotland lies on the convergence of the Atlantic Ocean and the North Sea, which provides both a wide range of ecological niches with regards to temperature and depth [44] and a source for both Northern and Southern bryozoan species [45]. There have been 7 published mineralogical studies [27,30,41,42,46–48] featuring bryozoan species which are present in the Scottish fauna. The earliest of these were conducted with titration [46,47], with the remainder being conducted with XRD, and, in a few cases, Raman spectroscopy [30]. These publications provided analyses of 41 species (n = 148), although specimens collected from Scottish waters were only used in the analysis of 5 species (n = 8).

Here we report comprehensively on the variation in bryozoan skeletal carbonate mineralogy from around Scotland and how it compares to other temperate regions. We quantify the effects of phylogeny on skeletal composition, and evaluate the extent to which environment plays a role in phenotypic expression, particularly within species.

## Materials and methods

### Sample collection, archiving, and preparation

This study is based on bryozoan species collected from Scottish waters. Material was taken from existing collections and field sampling in order to collect samples of as many species as possible. None of the specimens in this study are endangered or protected. Full details of all specimens can be found in Table A in S1 File.

The Bryozoa collections of the Natural History Museum (NHM) in London and the National Museum of Scotland (NMS) were searched for Scottish species with permission from the museum curators. 38 species were sourced from the NHM collection and 43 species were sourced from the NMS collection. The private collections of Dr Joanne Porter, Heriot-Watt University and Dr Jim Drewery, Marine Scotland (Rockall collection) were also investigated, resulting in a further 10 species. Field sampling was conducted by hand (mostly using SCUBA), in Scottish waters between 2010 and 2013. Sample collection localities ranged from 60.8⁰N in Shetland to 54.9⁰N in Stranraer, Scotland. Water depths ranged from intertidal to 35m but were mostly less than 20m. Permissions for field sampling were obtained where required (from private property or protected sites) and details can be found in Table B in S1 File. For field sampling from all other sites, which were neither private property nor protected, permission was not required.

Species were identified to species level under a dissection (stereo) microscope (Zeiss) using the monographs of Hayward and Ryland [49,50]. Taxonomy was corrected to match the World Register of Marine Species [51]. Samples were extracted from the tip of erect colonies and the growing edge of encrusting colonies. A minimum of 5 zooids were extracted for each sample. As far as possible, care was taken to ensure that no substrate (e.g. coralline algae) or epibionts were included within the sample as they could potentially contaminate results. Each specimen was examined under a microscope and any substrate or epibionts were carefully scraped away with the tip of a scalpel blade; no chemical cleaning or treatment was conducted on specimens as it can have an impact on mineralogy [52]. Rare, figured, type or holotype specimens were not sampled but were analyzed whole using non-destructive micro-XRD. Vouchers for all specimens were retained, and reference specimens for each species sampled have been accessioned to the collections of the NHM, London.

Data for an additional 508 specimens of 4 Scottish species were included from scientific publications [15,52].

### Skeletal mineralogy analysis

The majority of mineralogical analyses were conducted at the Imaging and Analysis Centre (NHM London) using semi-quantitative X-ray diffractometry (XRD) following methods described in Loxton et al. [14]. The XRD instrument used was a high-precision Nonius XRD with a position-sensitive detector and cobolt generated X-rays. Compositional information from XRD analysis is considered accurate to within 2% on a well-calibrated instrument [20].

Additionally, some rare, figured, type or holotype samples, as identified by underlining in Table 1, were analysed whole using non-destructive micro-XRD. Qualitative phase identification and mineralogical analyses of whole specimens were conducted at the NHM using Micro-XRD with a GeniX High Flux Beam Delivery System and an INEL 120⁰ position-sensitive-detector. FOX2D mirror optics (XENOCS) focussed the X-ray beam of copper radiation to a spot size of 230µm. Protruding and flat skeletal surfaces of the specimens were targeted using an AxioCam MRc5 microscope camera. The error associated with this method is higher than with traditional XRD due to potential non-random orientation of crystallites in calcified skeletons and minor sample displacement. In this study uncertainties were estimated to be approximately 10% based on the duplicate analysis of the same sample using both micro-XRD and conventional XRD. Both XRD and micro-XRD instruments were calibrated daily using pure silica (Si) and silver behenate (AgC_22_H_43_O_2_) on a quartz substrate [53,54].

**Table 1:** Skeletal carbonate mineralogy of 154 species of bryozoans. See Table A in S1 File or source literature [15, 52] for full details of each specimen.

Species Bold = first analysis of species Underline = first analysis of genus	No. of specimens (* = Micro XRD)	Wt.% MgCO_3_ in calcite. Mean if >1 specimen, Range (mean)	Wt% calcite range (mean)
***Aetea anguina*** (Linnaeus, 1758)	1*	7.9	100
***Aetea sica*** (Couch, 1844)	1*	-	100
***Aetea truncata*** (Landsborough, 1852)	1*	-	100
***Alderina imbellis*** (Hincks, 1860)	1	6.1	100
***Amphiblestrum auritum*** (Hincks, 1877)	1	5.6	100
*Amphiblestrum flemingii* (Busk, 1854)	1	3.6	100
***Amphiblestrum solidum*** (Packard, 1863)	1	7.2	100
***Anarthropora*** ***monodon*** (Busk, 1860)	3	-	0–0 (0)
***Annectocyma major*** (Johnston, 1847)	1*	6.4	100
***Beania mirabilis*** Johnston, 1840	1	7.2	100
***Bicrisia abyssicola*** Kluge, 1962	1	2.4	100
***Bicellariella*** ***ciliata*** (Linnaeus, 1758)	1	5.0	82
***Bicellarina*** ***alderi*** (Busk, 1859)	1	2.8	57
*Bugula neritina* (Linnaeus, 1758)	1	5.1	100
***Bugulina avicularia*** (Linnaeus, 1758)	1	4.8	100
***Bugulina flabellata*** (Thompson, in Gray, 1848)	1	3.9	58
***Bugulina fulva*** (Ryland, 1960)	1	6.6	100
***Bugulina simplex*** (Hincks, 1886)	1	3.9	49
***Bugulina turbinata*** (Alder, 1857)	1	2.2	100
***Buskea dichotoma*** (Hincks, 1862)	1	4.5	100
***Buskea nitida*** Heller, 1867	1	4.6	100
*Caberea ellisii* (Fleming, 1814)	1	4.9	100
*Callopora craticula* (Alder, 1856)	1	4.3	100
***Callopora dumerilii*** (Audouin, 1826)	1	7.5	100
*Callopora lineata* (Linnaeus, 1767)	1	5.2	83
***Callopora rylandi*** Bobin and Prenant, 1965	1	6.9	100
*Cauloramphus spiniferum* (Johnston, 1832)	1	3.8	85
***Cellaria fistulosa*** (Linnaeus, 1758)	16	3.5–7.1 (5.1)	100–100 (100)
*Cellaria salicornioides* Lamouroux, 1816	1	3.7	100
***Cellaria sinuosa*** (Hassall, 1840)	1	3.9	92
*Cellepora pumicosa* (Pallas, 1766)	1	4.7	89
*Celleporella hyalina* (Linnaeus, 1767)	1	1.7	100
*Celleporina caliciformis* (Lamouroux, 1816)	1	4.9	100
***Celleporina pygmaea*** (Norman, 1868)	1	7.2	100
*Carbasea carbasea* (Ellis and Solander, 1786)	1	6.3	100
***Chartella barleei*** (Busk, 1860)	1	9.6	28
*Chartella papyracea* (Ellis and Solander, 1786)	1	9.0	100
*Chorizopora brongniartii* (Audouin, 1826)	1	5.5	100
***Conopeum reticulum*** (Linnaeus, 1767)	1	5.0	100
*Conopeum seurati* (Canu, 1928)	1	9.1	95
***Coronopora*** ***truncata*** (Fleming, 1828)	1	7.8	100
*Cradoscrupocellaria reptans* (Linnaeus, 1758)	3	3.8–6.8 (5.1)	65–94 (82)
*Cribrilina annulata* (O. Fabricus, 1780)	1	5.0	89
*Cribrilina cryptooecium* Norman, 1903	1	2.9	100
***Cribrilina punctata*** (Hassall, 1841)	1	5.0	100
***Crisia aculeata*** Hassall, 1841	1	4.8	90
***Crisia denticulata*** (Lamarck, 1816)	1	3.3	100
*Crisia eburnea* (Linnaeus, 1758)	2	3.3–3.4 (3.3)	100
***Crisia ramosa*** Harmer, 1891	1	2.3	100
***Crisidia*** ***cornuta*** (Linnaeus, 1758)	1	5.0	100
***Crisularia plumosa*** (Pallas, 1766)	1	7.4	100
***Crisularia purpurotincta*** (Norman, 1868)	1	3.9	89
*Cryptosula pallasiana* (Moll, 1803)	1	4.6	59
***Cylindroporella tubulosa*** (Norman, 1868)	1*	7.0	100
*Dendrobeania murrayana* (Bean, in Johnston, 1847)	1	4.7	100
***Diplosolen obelia*** (Johnston, 1838)	1*	5.8	100
***Disporella hispida*** (Fleming, 1828)	1	5.2	100
***Doryporellina*** ***reticulata*** (Ryland, 1963)	1*	7.2	
***Einhornia crustulenta*** (Pallas, 1766)	1	4.1	100
***Electra monostachys*** (Busk, 1854)	1	7.3	91
*Electra pilosa* (Linnaeus, 1767)	14	6.8–9.9 (8.6)	53–100 (80.7)
***Entalophoroecia*** ***deflexa*** (Couch, 1842)	1	4.5	100
***Escharella abyssicola*** (Norman, 1869)	1	7.5	94
*Escharella immersa* (Fleming, 1828)	147^a^	4.3–6.9(5.7)	25-99(69.1)
***Escharella labiosa*** (Busk, 1856)	1*	5.8	100
***Escharella laqueata*** (Norman, 1864)	1*	6.0	100
***Escharella octodentata*** (Hincks, 1880)	1	7.3	100
*Escharella variolosa* (Johnston, 1838)	1	6.6	88
***Escharella ventricosa*** (Hassall, 1842)	1	5.6	100
***Escharina alderi*** (Busk, 1856)	1	7.0	100
***Escharina dutertrei haywardi*** Zabala, Maluquer and Harmelin, 1993	1	7.4	100
***Escharina johnstoni*** (Quelch, 1884)	1*	7.2	100
*Escharoides coccinea* (Abildgaard, 1806)	1	3.3	64
***Escharoides mamillata*** (Wood, 1844)	1	7.0	100
*Eucratea loricata* (Linnaeus, 1758)	1	7.7	85
***Eurystrotos*** ***compacta*** (Norman, 1866)	1	6.3	100
*Fenestrulina malusii* (Audouin, 1826)	1	2.8	88
***Filicrisia geniculata*** (Milne Edwards, 1838)	1	3.9	86
*Flustra foliacea* (Linnaeus, 1758)	97^b^	6.2–13.5(9.4)	100
***Haplopoma graniferum*** (Johnston, 1847)	1	3.1	75
*Haplopoma impressum* (Audouin, 1826)	1	1.8	100
***Haplopoma planum*** Ryland, 1963	1	6.1	100
***Haplopoma sciaphilum*** Silén and Harmelin, 1976	1*	7.5	
***Hemicyclopora*** ***polita*** (Norman, 1864)	1	7.3	100
***Herentia*** ***hyndmanni*** (Johnston, 1847)	1*	6.2	100
*Hippoporina pertusa* (Esper, 1796)	1*	6.3	100
***Hippothoa divaricata*** Lamouroux, 1821	1*	-	0
***Hornera lichenoides*** (Linnaeus, 1758)	1	5.6	94
Idmidronea atlantica *(Forbes*, *in Johnston*, 1847)	1	3.8	100
***Lagenipora lepralioides*** (Norman, 1868)	1*	5.2	100
***Larnacicus*** ***corniger*** (Busk, 1859)	1	7.7	94
***Lepraliella hippopus*** (Smitt, 1867)	1	7.7	96
***Marguetta*** ***lorea* (**Alder, 1864)	1	8.5	100
***Megapora*** ***ringens*** (Busk, 1856)	1	6.5	44
*Membranipora membranacea* (Linnaeus, 1767)	39	0-0(0)	0–0 (0)
*Membraniporella nitida* (Johnston, 1838)	140^a^	2.2–7.9(6.2)	100
*Microporella ciliata* (Pallas, 1766)	146^a^	4.5–8.7(6.9)	34-99(77.2)
***Neolagenopora*** ***eximia*** (Hincks, 1860)	1*	5.0	100
***Notoplites jeffreysii*** (Norman, 1868)	1	5.9	13
***Omalosecosa*** ***ramulosa*** (Linnaeus, 1767)	15	4.8–13.6 (9.8)	91–100 (98.2)
***Oncousoecia*** ***diastoporides*** (Norman, 1869)	1	6.9	87
***Oncousoecia*** ***dilatans*** (Johnston, 1847)	1	7.8	100
***Oshurkovia littoralis*** (Hastings, 1944)	1	6.0	48
***Palmicellaria elegans*** Alder, 1864	1*	7.4	100
***Palmiskenea*** ***skenei*** (Ellis and Solander, 1786)	1	5.9	100
***Parasmittina trispinosa*** (Johnston, 1838)	1	7.7	80
***Pentapora fascialis*** (Pallas, 1766)	1	8.4	54
***Phaeostachys*** ***spinifera*** (Johnston, 1847)	1	5.7	63
***Plagioecia patina*** (Lamarck, 1816)	1	6.2	100
***Porella alba*** Nordgaard, 1906	1	4.0	94
***Porella compressa*** (J. Sowerby, 1805)	1	8.2	100
***Porella concinna*** (Busk, 1854)	2	8.1–8.2 (8.1)	99–100 (99.5)
***Porella laevis*** (Fleming, 1828)	1	10.2	100
***Porella struma*** (Norman, 1868)	1*	5.9	100
***Pseudoflustra virgula*** Hayward, 1994	1	5.6	100
***Puellina innominata*** (Couch, 1844)	1	8.8	100
***Pyripora catenularia*** (Fleming, 1828)	1	8.7	90
***Ragionula*** ***rosacea*** (Busk, 1856)	1*	7.2	100
***Ramphonotu******s minax* (**Busk, 1860)	1	6.9	100
***Reteporella beaniana*** (King, 1846)	1	8.5	100
***Reteporella incognita*** Hayward and Ryland, 1996	1	8.4	100
***Reteporella watersi*** (Nordgaard, 1907)	1	5.8	87
***Rosseliana rosselii*** (Audouin, 1826)	1*	6.5	100
***Schizomavella (Schizomavella) auriculata*** (Hassall, 1842)	1	5.2	27
***Schizomavella (Schizomavella) linearis*** (Hassall, 1841)	1	6.4	1
***Schizoporella japonica*** Ortmann, 1890	19	3.9–7.4 (5.3)	19–86 (56.9)
***Schizoporella patula*** Hayward and Ryland, 1995	2	7.6–8.1 (7.9)	1–13 (7)
*Schizoporella unicornis* (Johnston, in Wood, 1844)	1	6.0	59
***Scruparia*** ***ambigua*** (d’Orbigny, 1841)	1	8.1	100
***Scruparia*** ***chelata*** (Linnaeus, 1858)	1	7.9	100
*Scrupocellaria scrupea* Busk, 1852	1	1.4	100
***Scrupocellaria scruposa*** (Linnaeus, 1758)	5	3.3–6.3 (4.6)	57–100 (91.4)
*Securiflustra securifrons* (Pallas, 1766)	1	9.8	91
*Setosella vulnerata* (Busk, 1860)	1	-	0
***Smittina bella*** (Busk, 1860)	1*	-	0
***Smittina crystallina*** (Norman, 1867)	1	8.1	67
***Smittoidea marmorea*** (Hincks, 1877)	1*	-	0
***Smittoidea reticulata*** (MacGillivray, 1842)	1	6.3	16
***Stigmatoechos*** ***violacea*** (M. Sars, 1863)	1	7.4	100
*Stomacrustula cruenta* (Busk, 1854)	1*	9.0	-
***Stomacrustula sinuosa*** (Busk, 1860)	1*	6.5	-
***Stomatopora*** ***gingrina*** Jullien, 1882	1	7.2	93
*Tegella unicornis* (Fleming, 1828)	1	3.6	100
***Temachia*** ***microstoma*** (Norman, 1864)	1*	6.3	100
*Tervia irregularis* (Meneghini, 1844)	1	8.0	100
*Tessaradoma boreale* (Busk, 1860)	1	6.4	100
***Tricellaria inopinata*** d’Hondt and Occhipinti Ambrogi, 1985	1	2.4	100
*Tricellaria ternata* (Ellis and Solander, 1786)	1	5.0	100
***Tubulipora liliacea*** (Pallas, 1766)	2	2.6–2.7 (2.6)	97–100 (98.5)
***Tubulipora penicillata*** (O. Fabricius, 1780)	1	4.7	100
*Tubulipora phalangea* Couch, 1844	1	1.9	100
***Tubulipora plumosa*** Thompson, in Harmer, 1898	1	1.0	100
***Turbicellepora avicularis*** (Hincks, 1860)	1	9.0	100
***Turbicellepora boreale*** Hayward and Hansen, 1999	1	7.0	100
N	790	147	150
Min	1	0	0
Mean	5	6	87
Max	147	10	100
Range	146	9	100

^a^ includes analyses from Loxton et al., 2014 [15]–*Membraniporella nitida* (n = 139), *Microporella ciliata* (n = 145) and *Escharella immersa* (n = 146)

^b^ includes analyses from Loxton et al., 2017 [52]–*Flustra foliacea* (n = 78)

### Statistics and data analysis

Wt% MgCO_3_ in calcite data for all species was tested for normality using Anderson-Darling normality tests. Criteria for parametric *posthoc* testing were not met due to unequal sample sizes and heterogeneous variance between datasets.

Phylogenetic data and branch lengths were taken from the publication of Waeschenbach et al. [55]. Branch length of the phylogeny indicates the number of substitutions per site based on Bayesian multi-gene analysis of a concatenated 7-gene dataset *(ssrDNA*, *lsrDNA*, *rrnL*, *rrnS*, *cox1*, *cox3*, *cytb*) constructed using BEAST under the random logical clock and GTR+I+G model [55]. The phylogeny was input into the R statistical language [56] using Newman coding. Kembel’s [57] methodology for Comparative Phylogenetic Methods was followed. Comparative phylogenetic diversity and phylogenetic distance between cyclostomes and cheilostomes were calculated using the “pd” and “cophenetic” functions in the Picante package for R [58,59]. Blomberg’s K [60] values for cyclostomes, cheilostomes and all Scottish bryozoans were calculated as a measure of underlying phylogenetic signal in the traits of wt% MgCO_3_ in calcite and wt% calcite using the “multiPhylosignal” function in the Picante package for R [58,59]. Blomberg’s K was also calculated as a measure of underlying phylogenetic signal for the trait of morphology.

To elucidate differences between taxonomic, spatial, evolutionary and ecological groupings, non-parametric Mann-Whitney U-tests were carried out. Data from three temperate regional studies (Scotland, this study; Chile, [24]; New Zealand, [27]) were each analysed using a generalised linear model (GLM) ANOVA. The factor used was region (fixed) and the response wt% MgCO_3_ in calcite. Criteria for parametric *posthoc* testing were not met due to unequal sample sizes between datasets, therefore potential differences between regions were elucidated by Mann-Whitney *Post-hoc* testing.

## Results

The skeletons of 790 bryozoan specimens from a total 154 species from temperate Scottish waters were analysed or collated from literature (Table 1) and had a mean wt% calcite of 84. The most common mineralogy type was 100% calcite; 93 species (60%) were formed entirely from calcite. Bimineralic species, containing a mixture of calcite and aragonite polymorphs, were the second most common mineralogy type. Fifty-five species, or 36% of those tested had this type of mineralogy and the proportion of the two types was spread fairly evenly across the possible proportional range, from 1% to 99% calcite. Entirely aragonitic species were the least common with just 6 species (4%) featuring this type of mineralogy.

All specimens were analysed for wt% MgCO_3_ in calcite, which ranged from 0 to 14, with a mean of 6. The majority (69%) of species featuring calcite were classed as intermediate-Mg calcite (4–8 wt% MgCO_3_). A further 18% formed low-Mg calcite (0–4 wt% MgCO_3_) and the remaining 12% of species featured high-Mg calcite (8–12 wt% MgCO_3_).

See Table A in S1 File for full specimen details.

**Fig 1:**
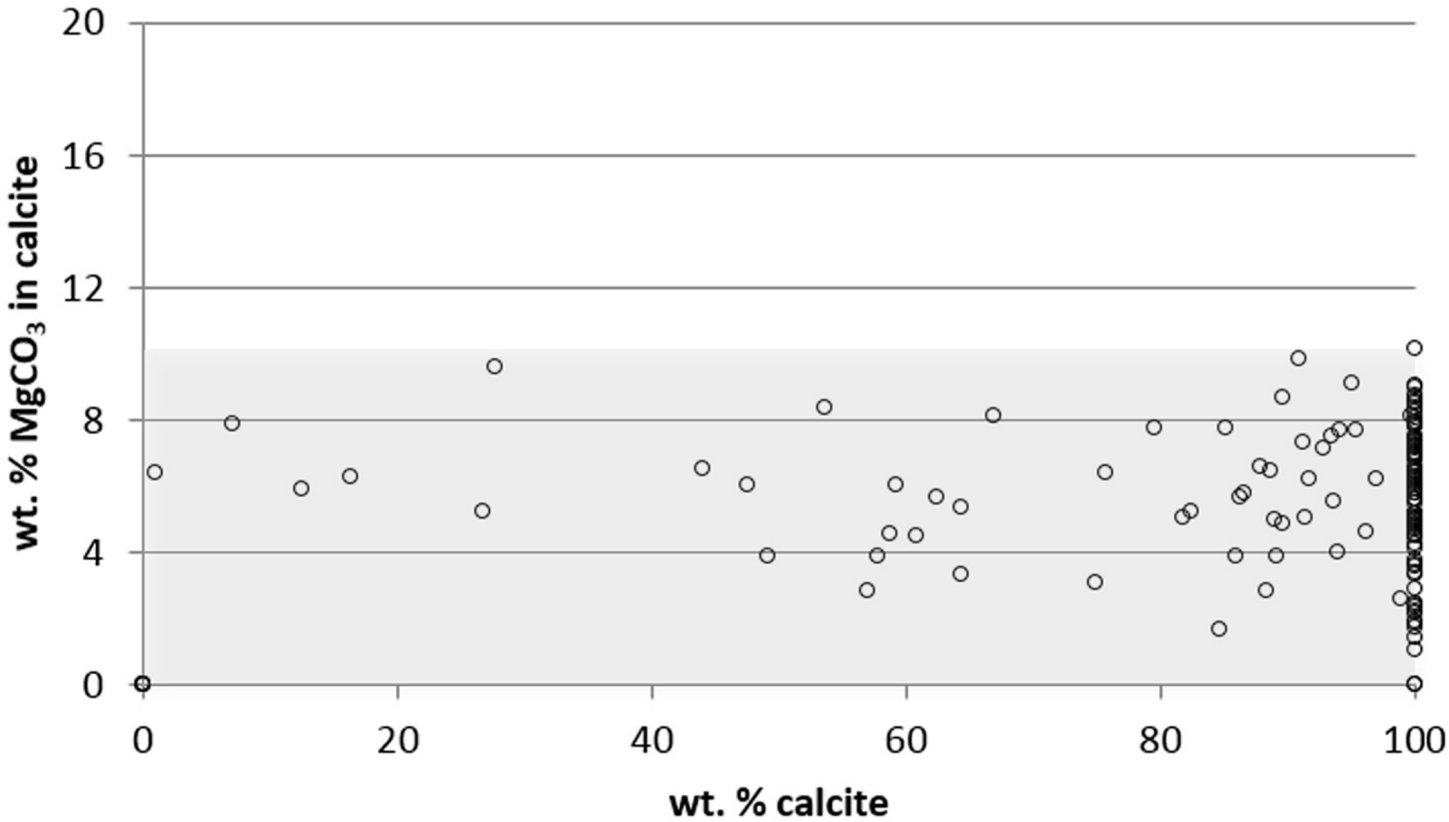
The biomineral space occupied by Scottish bryozoan species. Skeletal carbonate mineralogy of 154 species of Scottish marine bryozoans. Shaded box indicates maximum biomineral space occupied by the phylum in Scotland (42% of the total available biomineral space).

Range of carbonate mineralogy in a group of bryozoan specimens is described as biomineral “space”, a term introduced by Smith et al [27]. Biomineral space is the area described by the ranges of wt% MgCO_3_ in calcite and wt% calcite for a particular species of group of species. It is usually expressed as a percentage of the possible space available for biomineralization (0–22 wt% MgCO_3_ in calcite and 0–100 wt% calcite, a possible space of 2178wt%^2^). The biomineral space for Scottish bryozoans is shown in Fig 1 with Scottish species covering 42% of the total available mineral space.

### Comparison to other regions

Table 2 compares the mineralogical profile of Scottish bryozoan species to those reported from other temperate regions, New Zealand and Chile, and to the neighbouring Arctic. This shows consistency among the mineralogical means reported from the temperate regions of Scotland, New Zealand and Chile although variation can be found among the ratios of species found in different mineralogical categories. The Arctic was the only region to show nearly 100% calcite across all species and lower incorporation of MgCO_3_ in calcite than was found in any of the temperate regions.

**Table 2:** Comparison of regional studies of bryozoan mineralogy. The ABC (aragonitic, bimineralic, calcitic) index quantifies the ratio of bryozoan species with particular mineralogies as first introduced by Borszcz et al [61].

	Scotland (this study, incl. data from [15,52])	New Zealand [43]	Chile [24]	Arctic [20]
Number of specimens	790	412	93	149
Number of species	154	49	23	76
Number of families	50	29	15	31
Distribution (latitude)	55–60°N	25–52°S	42–52°S	69–79°N
ABC index (aragonitic, biomineralic, calcitic ratio)	6:55:93	3:13:33	2:2:19	0:3:73
Aragonite: Bimineral: Calcite (% of species)	4:36:60	6:27:67	9:9:82	0:4:96
HMC: IMC: LMC (% of species)	6: 65: 29	0:86:14	18:57:25	0:57:43
Mean wt% calcite	84	85	84	100
Mean wt% MgCO_3_ in calcite	5.1	4.3	5.5	4.0

There is a statistical difference in species wt% Mg in calcite between Scotland, New Zealand and Chile (ANOVA, F = 3.12, P = 0.046*). *Post-hoc* Mann-Whitney analysis shows Scotland to be statistically different to New Zealand (P = 0.030*) but not Chile and no statistical difference between Chile and New Zealand. Wt % MgCO_3_ in calcite in Scottish species (n = 154) is statistically different (Mann-Whitney U-test, P<0.001*) to those from its geographical neighbour, the Arctic (n = 76) [20].

### Taxonomic patterns

#### 1. Class/Order

125 species of Scottish cheilostomatous Bryozoa were found to contain a wide range of mineralogical compositions. 57% (n = 73) of species were found to be entirely calcitic with a further 39% (n = 50) featuring bimineralic mixing in some degree and only 5% (n = 6) found to be pure aragonite. The measured range for this group spanned the entire range from 0–100 wt% calcite, with a mean of 84 (n = 125). The majority (70%, n = 85) of species featured intermediate-Mg calcite (IMC) with the remaining species equally split between low-Mg calcite (LMC) and high-Mg calcite (HMC) (15%, n = 18 for both IMC and HMC). The mean of 6.0 wt% MgCO_3_ in calcite (n = 125) reflects the dominance of IMC in Scottish cheilostomatous species.

27 specimens of 25 cyclostomatous species were analysed with 20 (80%) found to be entirely calcitic. The remaining five species showed some aragonite with the measured range showing between 82 and 99 wt% calcite. The mean wt% calcite for this class in Scotland is thus 98.5. This mean value for the Order Cyclostomatida is significantly higher than the mean for the Order Cheilostomatida (mean = 84.3, n = 125) (Mann-Whitney U-test, P = 0.016*).

#### 2. Families

The specimens studied come from 47 families in the phylum Bryozoa, almost all (94%) of the Recent families reported to occur in Scotland. Many of these families are only represented by one or two species although some include as many as 14 species. Although some families contain only one analysed specimen and conclusions should, therefore, be approached with caution, the mean number of specimens analysed per family is 16, and the maximum is 180, allowing some generalizations to be made. Coverage of Scottish genera within analysed families range from 33–100% with a mean of 81% of Scottish genera included here.

#### 3. Species

**Fig 2:**
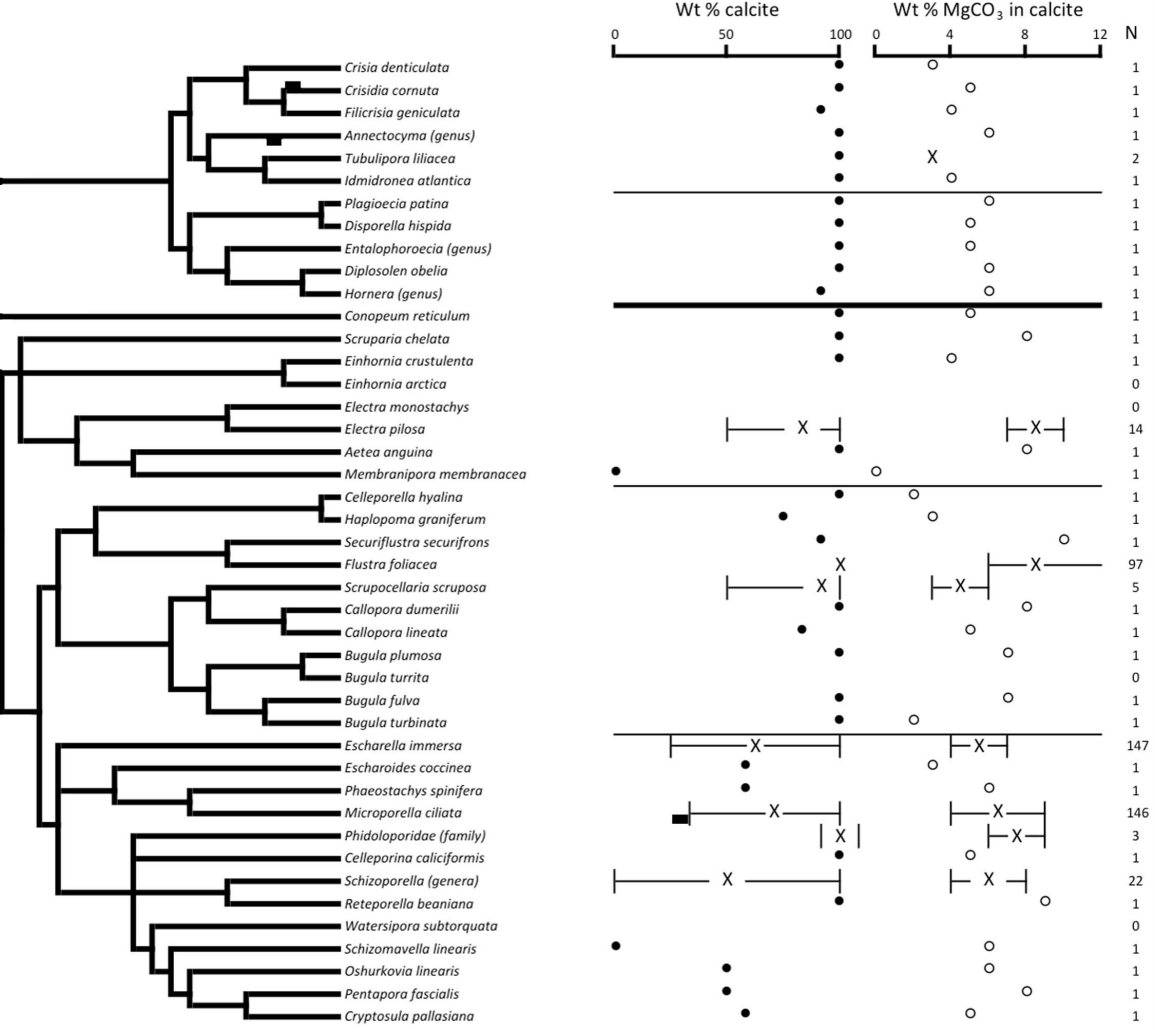
The phylogenetic distribution of the skeletal carbonate mineralogy of Scottish bryozoans. Phylogenetic tree adapted from Waeschenbach et al [55]. Crosses are means, with the range delineated by tails. Single measurements are represented by black (wt% MgCO_3_ in calcite) or hollow (wt% calcite) circles.

The phylogenetic position for 39 (11 cyclostomes and 28 cheilostomes) Scottish bryozoan species were extracted from the bryozoan phylogeny published by Waeschenbach et al. [55] (Fig 2). There is no phylogenetic signal for the mineralogical trait of Mg content in calcite (Blomberg’s K = 0.17, P = 0.264) although a strong and significant phylogenetic signal relating to calcite percentage was found (Blomberg’s K = 0.37, P = 0.022*). A similar analysis on the 28 cheilostomatous species for which phylogeny data is available revealed no phylogenetic signal associated with wt% MgCO_3_ in calcite (Blomberg’s K = 0.38, P = 0.25) but a statistically significant, strong phylogenetic signal associated with wt% calcite (Blomberg’s K = 0.82, P = 0.02*). Phylogenetic data was available for 11 cyclostome bryozoans and analysis of this revealed that there is no phylogenetic signal within mineralogy for either wt% MgCO_3_ in calcite (Blomberg’s K = 0.75, P = 0.2) or calcite (Blomberg’s K = 0.2, P = 0.99).

Most bryozoan species were only tested once, but nine common species had more than 10 replicates: *Cellaria fistulosa* (n = 16), *Electra pilosa* (N = 14), *Flustra foliacea* (n = 97), *Membranipora membranacea* (n = 39), *Omalosecosa ramuosa* (n = 15), *Escharella immersa* (n = 147), *Membraniporella nitida* (n = 140), *Microporella ciliata* (n = 146) and *Schizoporella japonica* (n = 19), allowing some consideration of within-species environmental plasticity.

*Cellaria fistulosa* (n = 16) consistently exhibited two phases of calcite within its skeleton; both phases were reported in all specimens in an approximate ratio of 1:1. The first phase of LMC has a mean of 1.9 wt% MgCO_3_ in calcite (range 0.5–3.7), while the second IMC phase showed a similar range of variation around a mean of 7.6 wt% MgCO_3_ in calcite (range 5.9–9.9). *Electra pilosa* (n = 14) is also bimineralic, with a mean aragonite content of 19.3% (range 47%-0%) and the dominant HMC varying around a mean of 8.6 wt% MgCO_3_ in calcite (range 6.8–9.9). *Flustra foliacea* (n = 97) was found to be a variable species with mean HMC of 9.4 wt% MgCO_3_ in calcite and a range of 7.3(range 6.2–13.5). *Membranipora membranacea* (n = 39), in contrast, had absolutely no mineralogical variability with a consistent 100% aragonite skeleton. *Omalosecosa ramulosa* (n = 15) had the most variable calcite of all species analysed with calcite ranging from IMC to HMC and a mean of 9.8 wt% MgCO_s_ in calcite and a range of 8.8 (range 4.8–13.6). *Escharella immersa* (n = 147) is bimineralic with a mean wt% MgCO_3_ in calcite of 5.7 (range 4.3–6.9) and a mean wt% calcite of 69.1 (mean 25–99). *Membraniporella nitida* (n = 140) contained no aragonite and has a mean wt % MgCO_3_ in calcite of 6.2 (range 2.2–7.9). *Microporella ciliata* featured a mean of 6.9 wt% MgCO_3_ in calcite (range 4.5–8.7) and a mean wt% calcite of 77.2 (range 34–99). *Schizoporella japonica* was the only non-native species with multiple analyses in this study and exhibited a bimineralic skeleton with a high degree of both calcitic and aragonitic variation. The mean of 43% aragonite in the skeleton varied widely (range 14–81%), and the IMC had a mean of 5.3 wt% MgCO_3_ in calcite (range 3.9–7.4).

## Discussion

### Mineralogy of the phylum Bryozoa

This regional study of Scottish bryozoans contributes 282 measurements, and collates a further 508 measurements, of 154 species to existing knowledge of bryozoan mineralogy, including 110 species never before measured. These data increase the number of genera studied by 26, and the number of families by 4. In terms of both scope and environment, it is a Northern Hemisphere equivalent to published studies of New Zealand bryozoans [43] and Chilean bryozoans [24]. In this study we compared the taxonomic patterns discerned here with these two Southern Hemisphere temperate communities, and for contrast, with the neighbouring Arctic community. Table 2 shows the remarkable consistency among the three temperate regions with Scotland, New Zealand and Chile featuring means of 5.1, 4.3 and 5.5 wt% MgCO3 in calcite and 84, 85, 84% calcite respectively. This dominance of IMC in bryozoan skeletons from temperate waters has been reported in previous publications investigating global [27] and regional [24,43] mineralogical patterns in the Bryozoa. Despite the similarity of the means, Scotland shows more variety in wt% MgCO_3_ in calcite than New Zealand or Chile. This may be accounted for by the greater number of species presented in this study compared to the other regional studies (Table 2). The wide range of mineralogical variety and the solid presence of aragonite, LMC and HMC in Scottish bryozoan fauna may also be a combined reflection of the inclusion of Polar/Boreal and Lusitanian species in the Scottish fauna, due to Scotland’s open geographical situation, and the diverse range of habitats and seasonal conditions which it offers.

Arctic species feature lower wt% MgCO_3_ in calcite and less aragonite that Scottish species. Polar species feature slow growth rates [16, 62, 63] caused by low nutrient levels, low temperature and increased seasonality [16,64–66]. Links between mineralogy and growth rate have been shown with slower animals generally depositing less wt% MgCO_3_ in calcite [67]. In addition the low temperatures and intrinsically low saturation of carbonate ions [68] in Polar waters means that deposition of calcite is favoured over aragonite [18,20,68]. The Arctic fauna features a few endemic species but predominantly consists of species with Pacific or Atlantic origin which entered Arctic waters following the last glacial maximum approximately 25 thousand years ago [69]. Arctic species exhibit a lower mean wt% MgCO_3_ in calcite than Scotland, showing some adaptation to the Polar environment [18], although this mean is closer to that exhibited in Scotland than the mean for Antarctica [42], and possibly reflects the relatively young bryozoan fauna in the Arctic.

### Mineralogy of classes/orders in the Bryozoa

Previous studies, including Smith et al., 2006 [27] and Boardman and Cheetham, 1987 [70], have found cyclostomes to be almost entirely calcitic. Boardman and Cheetham went as far as to state “All stenolaemates have calcareous skeletons. All skeletons are calcitic except for one reportedly aragonitic species from the Triassic.” [70] Cyclostomatida is the only extant order of the five comprising the Class Stenolaemata; it is only Recent specimens from this order which have been analysed in this Scottish study. Like Smith et al. [27] we find Boardman and Cheetham’s statement to be mostly accurate although, like Smith et al. [27], we also found trace amounts of aragonite in a few cases. Cheilostomes were found to be much more variable than cyclostomes and further investigation is recommended into the evolutionary radiation of cheilostomes and how that may have contributed to their greater biomineral space.

### Mineralogy of families in the bryozoa

Smith et al. [27] surmised that bryozoan families fall into three general groups; those containing mostly aragonite, those containing mixed mineralogy and those containing mostly calcite. In general the data presented in Tables 1 and 3 concurs with this, although the distribution of Scottish species within these categories. (4% mostly aragonite, 32% mixed mineralogy, 64% mostly calcite) varies slightly from the Global data presented by Smith et al. [27] (5% mostly aragonite, 20% mixed mineralogy, 75% mostly calcite).

**Table 3:** Mineralogical characteristics of 50 bryozoa families from Scotland. The FAD stage details the first appearance datum, which is the first (oldest) appearance of the family in the geological record; it is reported here on the geologic time scale. The age at top (Ma) details how many million years ago these geological time periods were where they commenced.

Family	Genera	Species	n	Mean wt.% calcite	Mean wt.% MgCO_3_ in calcite	FAD stage	Age at top (Ma)
**Aeteidae**	1	3	3	100.0	7.9	Pliensbachian*	190
**Annectocymidae**	1	1	1	100.0	6.4	Albian***	110
**Antroporidae**	1	1	1	100.0	6.5	Maastrichtian**	65
**Beaniidae**	1	1	1	100.0	7.5	Bartonian**	37
**Bitectiporidae**	3	4	4	45.3	6.6	Ypresian**	49
**Bryocryptellidae**	3	8	9	99.2	7.2	Ypresian**	49
**Bugulidae**	4	11	11	85.0	4.6	Recent**	0
**Calloporidae**	7	12	12	92.6	5.4	Albian**	99
**Candidae**	4	7	13	87.5	4.2	Maastrichtian**	65
**Cellariidae**	1	3	18	97.3	5.3	Santonian**	83.5
**Celleporidae**	7	10	24	98.6	6.2	Priabonian**	33.7
**Chaperiidae**	1	1	1	5.9	7.7	Maastrichtian**	65
**Chorizoporidae**	1	1	1	100.0	5.5	Servallian**	11.2
**Cribrilinidae**	3	5	5	97.8	4.8	Cenomanian**	93.5
**Crisiidae**	4	7	8	96.5	3.6	Maastrichtian**	65
**Cryptosulidae**	1	1	1	58.8	4.6	Tortonian**	7.1
**Diaperoeciidae**	1	1	1	100.0	4.5	Hauterivian**	127
**Diastoporidae**	1	1	1	100.0	5.8	Pliensbachian**	190
**Doryporellidae**	1	1	1		7.2	Albian***	110
**Electridae**	3	4	16	89.2	6.6	Tithonian*	163.5
**Escharinidae**	3	5	152	92.5	6.7	Ypresian*	49
**Eucrateidae**	1	1	1	85.2	7.7	Recent**	0
**Exochenellidae**	1	1	3	0.0	0.0	Ypresian*	49
**Exochellidae**	1	2	2	82.2	5.1	Santonian*	83.5
**Flustridae**	4	5	101	83.7	8.7	Recent**	0
**Haplopomidae**	1	4	4	91.6	4.6	Lutetian**	90
**Hippoporidridae**	0	0	0			Chattian*	23.03
**Hippothoidae**	2	2	2	50.0	1.7	Coniacian**	85.8
**Horneridae**	1	1	1	93.7	5.6	Barremian**	121
**Lacernidae**	0	0	0			Priabonian**	33.7
**Lepraliellidae**	1	1	1	95.5	7.7	Santonian**	83.5
**Lichenoporidae**	2	2	2	100.0	6.5	Cenomanian**	93.5
**Membraniporidae**	2	3	180	65.1	7.1	Priabonian**	33.7
**Microporellidae**	2	2	147	74.6	3.7	Aquitanian**	20.5
**Microporidae**	0	0	0				
**Oncousoeciidae**	2	3	3	100.0	7.0	Sinemurian*	199.3
**Phidoloporidrae**	1	3	3	95.6	7.6	Dunian**	61
**Plagioeciidae**	1	1	1	100.0	6.2	Pliensbachian**	190
**Romancheinidae**	4	10	10	96.8	6.5	Campanian**	71.3
**Schizoporellidae**	1	3	22	43.5	6.4	Ypresian**	49
**Scrupariidae**	1	2	2	100.0	8.0	Maastrichtian*	70
**Setosellidae**	1	1	1	0.0	0.0	Thanetian**	54.8
**Smittinidae**	5	7	7	51.8	6.5	Ypresian**	49
**Stigmatoechidae**	1	1	1	100.0	7.4	Camponian*	83.6
**Stomachetosellidae**	1	2	2		7.7	Maastrictian*	70
**Stomatoporidae**	1	1	1	92.8	7.2	Carnian*	235
**Terviidae**	1	1	1	100.0	8.0	Ypresian**	49
**Tessaradomidae**	1	1	1	100.0	6.4	Danian**	61
**Tubuliporidae**	2	5	6	99.8	2.8	Campanian**	71.3
**Umbonulidae**	1	1	1	47.6	6.0	Lutetian**	41.3

First appearance datum (FAD) taken from the following sources

* Taylor, 1993 [71]

**Smith et al., 2006 [27]

*** Taylor et al., 2009 [30]

Most families, where more than one specimen has been analysed, show some level of variation within their mineralogy. Some of the IMC families that exhibit little or no variation are Aetidae (n = 3), Licheniporidae (n = 2) and Oncousoeciidae (n = 3). There are no families that consistently produce LMC or HMC. Families which are exclusively aragonitic (Setosellidae (n = 1), Exochenellidae (n = 3) and those which are aragonite-dominated (Stomachetosellidae, n = 2; Schizoporellidae, n = 22; Chaperiidae, n = 1) are found to be a mixture of flustrinid and ascophoran cheilostomes. The most variable family studied from Scotland is the Membraniporidae (18.64% of potential mineralogical space, n = 41).

There is no statistically significant difference between wt% MgCO_3_ in calcite in families that appeared during aragonitic and calcitic seas (Table 3). Families that first appeared during periods of aragonitic seas (approx 50 Ma-Recent and during the Triassic), do however, almost exclusively feature intermediate to high Mg-calcite (Mean = 6.0, n = 21). This fits with the sea water chemistry of the time where Mg/Ca ratios were at their highest. Families forming LMC all evolved during times of calcitic seas where the Mg/Ca ratio in seawater was much lower, however more research is needed to confirm this potential pattern.

As has been highlighted by phylogenetic studies [55,72], there is a high degree of taxonomic uncertainty surrounding many bryozoan families which means that great care should be taken when applying generalisations at the family level. It should also be considered that many families are represented by low numbers of specimens in this dataset. Due to limited metadata in the case of some museum specimens, it has not been possible to consider variability, which may be caused by collection location, depth, season and developmental stage of individuals; all factors which are known to influence mineralogy.

### Phylomineralogy in Scottish bryozoan species

Evolutionary origin of different species can be quantified using phylogenetic trees that often use branch length to approximate genetic distance of a species from their next nearest neighbour and offer an alternative to the use of First Appearance Date (FAD). A relatively recent advance in mineralogical methodology is the analysis of skeletal mineralogy alongside phylogenetic position of taxa, also termed “phylomineralogy” [1]. In many mineralogical studies samples are taken from multiple bryozoan lineages, and therefore do not represent statistically independent samples [60,73,74]. Some of the mineralogical differences between different species may be due to the divergent evolutionary history of the species and taxa [75], as shown in Fig 2. With the increasing availability of phylogenetic data mineralogical patterns can be assessed for a “phylogenetic signal” which can be quantified using measures such as Blomberg’s K [60] and taken into account during subsequent analysis and discussion. Phylogenetic data can also be included in Bayesian computational tools [76] where a variable such as phylogeny can be assessed as a potential explanation for observed species traits. Examples of where this methodology has been applied to mineralogical traits of other taxa include Cairn et al.’s work on cnidarians [23] and recent publications by Smith et al on serpulids [77] and coralline algae [1]. To date no bryozoan mineralogical analyses have taken into account the phylogenetic signal, however, the recent publication of bryozoan phylogenies by Tsyganov-Bodounov et al. [78], Fuchs et al. in 2009 [79] and Waeschenbach et al in 2012 [55,72] allow this concept to be tested for Bryozoa for the first time.

### Mineralogical variation within species

Evolutionary/phylogenetic theories help to explain the differences in base mineralogy between taxonomic groups and species, however in all species skeletal variation continues between different specimens within the same species.

The two main controls which are exerted on bryozoan mineralogy within species are summarised by many authors as biological and environmental control [20,27,29,32,80–84], also known as “active” and “passive” control in some publications [85,86]. Biological or “active” control usually refers to factors such as astogeny, the thickening of secondary calcification in older zooids [20,21,43], although it could also be considered to include growth rates [16,18,17], breeding cycles, food availability [19], physiological “wellness”[87] and directed mineralization to confer a competitive advantage [21,88,89]. Environmental control, or “passive” control, suggests that skeletal mineralogy is driven by the seawater from which the bryozoan is forming its skeleton with little or no physiological involvement from the animal itself. The main environmental control usually discussed is temperature [14,15,20,29,80,90], with higher temperatures reported to drive higher Mg-calcite and aragonite deposition. Other environmental factors which have also been shown to influence mineralogy include salinity [47], depth [40,61], aragonite compensation depth (ACD) [91], Mg/Ca ratio in seawater [80,81,88,90,92] and general seawater chemistry [29,87]. Many of these environmental factors, such as temperature, salinity, Mg/Ca ratio and ACD vary with latitude and have resulted in reported correlations between latitude and skeleton mineralogy [20,43,93]. Depth may be an explanatory factor for some of the variation seen within this study as samples were often collected from varying depths. Patterns of variation have been previously shown in bryozoan studies of both Scottish and Arctic species [15,61].

A sub-group of cheilostomes that feature both a wide geographical range and a corresponding wide range in mineralogy are those that have become successful as non-native (alien) species. An example from this study is *Schizoporella japonica* a species which was first found in Scotland in 2011 [94] and has since been found to inhabit marinas and harbours across Scotland and further afield [95]. This species has been reported from Norway to Malaysia and has highly plastic skeletal mineralogy (Table 1). It could be that the wide latitudinal range, and the wide variety in seawater encompassed, explains the correspondingly wide range of wt% MgCO_3_ in calcite in skeletons of this species. Alternatively, it could be that mineralogical plasticity itself enables the species to survive in a greater range of habitats enabling it to increase its distribution and making it well suited to colonising new marine habitats.

A more likely explanation for the variability seen in non-native species, however, is misidentification and cryptic species. An example is *Schizoporella unicornis* which is a non-native species in a number of countries and has been widely reported in bryozoan mineralogical publications for its mineralogical plasticity [27,32,38,47,96,97] with authors using and recommending it as an ideal species for environmental correlations with mineralogy [32] and potential palaeoenvironmental interpretation [27]. Recent publications have highlighted that many records and museum specimens previously identified as *S*. *unicornis* are actually misidentified *S*.*japonica* [94,95], *S*.*errata S*.*dunkeri* or other *Schizoporella* species [98–100]. Similar studies have been recently conducted on invasive species of *Watersipora* [101,102] and *Bugula* [103] which also identified taxonomic misidentifications and cryptic speciation. With this taxonomic doubt cast on past distribution and mineralogical records of many non-native species, caution should be exercised when interpreting patterns relating mineralogy to distributional range.

## Conclusion

This study describes the mineralogy of 156 species of the phylum Bryozoa within Scotland, representing 79% of the species that inhabits Scotland, a greater number and proportion of extant species than any other regional study. Analysis of the skeletal composition of Scottish bryozoans shows that the group is statistically different from the neighbouring Arctic fauna but features a range of mineralogy comparable to other temperate regions around the world.

Analysis of the mineralogical composition of Scottish bryozoans shows that the group reflects both reported patterns in evolutionary/ genetically “pre-programmed” mineralogy superimposed by variation driven by a combination of environment and biological/physiological factors. In such a variable region as Scotland, open to biological and environmental influx from all directions, it is perhaps no surprise that bryozoans reflect this diversity in the wide range of mineralogy they present.

These data add to a growing database of bryozoan mineralogical analyses from around the world, but we also highlight that more study is needed for a better understanding of the influence of genetic/ evolutionary, environmental and biological factors at play in bryozoan mineralogy.

## Supporting information

S1 File Full sample data and results. This file contains two tables: Table A contains sample data; Table B specifies sampling sites and permissions.
(XLSX)
